# Genomic Classification and Individualized Prognosis in Multiple Myeloma

**DOI:** 10.1200/JCO.23.01277

**Published:** 2024-01-09

**Authors:** Francesco Maura, Arjun Raj Rajanna, Bachisio Ziccheddu, Alexandra M. Poos, Andriy Derkach, Kylee Maclachlan, Michael Durante, Benjamin Diamond, Marios Papadimitriou, Faith Davies, Eileen M. Boyle, Brian Walker, Malin Hultcrantz, Ariosto Silva, Oliver Hampton, Jamie K. Teer, Erin M. Siegel, Niccolò Bolli, Graham H. Jackson, Martin Kaiser, Charlotte Pawlyn, Gordon Cook, Dickran Kazandjian, Caleb Stein, Marta Chesi, Leif Bergsagel, Elias K. Mai, Hartmut Goldschmidt, Katja C. Weisel, Roland Fenk, Marc S. Raab, Fritz Van Rhee, Saad Usmani, Kenneth H. Shain, Niels Weinhold, Gareth Morgan, Ola Landgren

**Affiliations:** ^1^ Myeloma Division, Sylvester Comprehensive Cancer Center, University of Miami, Miami, FL; ^2^ Heidelberg Myeloma Center, Department of Medicine V, University Hospital Heidelberg, Heidelberg, Germany; ^3^ Clinical Cooperation Unit (CCU) Molecular Hematology/Oncology, German Cancer Research Center (DKFZ), Heidelberg, Germany; ^4^ Department of Epidemiology and Biostatistics, Memorial Sloan Kettering Cancer Center, New York, NY; ^5^ Myeloma Service, Department of Medicine, Memorial Sloan Kettering Cancer Center, New York, NY; ^6^ Myeloma Research Program, New York University Langone, Perlmutter Cancer Center, New York, NY; ^7^ Division of Hematology Oncology, Melvin and Bren Simon Comprehensive Cancer Center, Indiana University, Indianapolis, IN; ^8^ Department of Malignant Hematology, Moffitt Cancer Center, Tampa, FL; ^9^ Aster Insights, Tampa, FL; ^10^ Department of Biostatistics & Bioinformatics, Moffitt Cancer Center & Research Institute, Tampa, FL; ^11^ Department of Cancer Epidemiology, Moffitt Cancer Center, Tampa, FL; ^12^ Hematology Unit, Fondazione IRCCS Ca' Granda Ospedale Maggiore Policlinico, Milan, Italy; ^13^ Department of Oncology and Onco-Hematology, University of Milan, Milan, Italy; ^14^ Freeman Hospital, The Newcastle Upon Tyne Hospitals NHS Foundation Trust, Newcastle, United Kingdom; ^15^ The Institute of Cancer Research, London, United Kingdom; ^16^ Leeds Cancer Research UK Clinical Trials Unit, Leeds Institute of Clinical Trials Research, University of Leeds, Leeds, United Kingdom; ^17^ Division of Hematology/Oncology, Mayo Clinic Arizona, Scottsdale, AZ, USA; ^18^ Department of Oncology, Hematology and Blood and Marrow Transplant, University Medical Center Hamburg-Eppendorf, Hamburg, Germany; ^19^ Department of Hematology, Oncology and Clinical Immunology, University-Hospital Duesseldorf, Duesseldorf, Germany; ^20^ Myeloma Institute for Research & Therapy, University of Arkansas for Medical Sciences, Little Rock, AR

## Abstract

**PURPOSE:**

Outcomes for patients with newly diagnosed multiple myeloma (NDMM) are heterogenous, with overall survival (OS) ranging from months to over 10 years.

**METHODS:**

To decipher and predict the molecular and clinical heterogeneity of NDMM, we assembled a series of 1,933 patients with available clinical, genomic, and therapeutic data.

**RESULTS:**

Leveraging a comprehensive catalog of genomic drivers, we identified 12 groups, expanding on previous gene expression–based molecular classifications. To build a model predicting individualized risk in NDMM (IRMMa), we integrated clinical, genomic, and treatment variables. To correct for time-dependent variables, including high-dose melphalan followed by autologous stem-cell transplantation (HDM-ASCT), and maintenance therapy, a multi-state model was designed. The IRMMa model accuracy was significantly higher than all comparator prognostic models, with a c-index for OS of 0.726, compared with International Staging System (ISS; 0.61), revised-ISS (0.572), and R2-ISS (0.625). Integral to model accuracy was 20 genomic features, including 1q21 gain/amp, del 1p, *TP53* loss, *NSD2* translocations, APOBEC mutational signatures, and copy-number signatures (reflecting the complex structural variant chromothripsis). IRMMa accuracy and superiority compared with other prognostic models were validated on 256 patients enrolled in the GMMG-HD6 (ClinicalTrials.gov identifier: NCT02495922) clinical trial. Individualized patient risks were significantly affected across the 12 genomic groups by different treatment strategies (ie, treatment variance), which was used to identify patients for whom HDM-ASCT is particularly effective versus patients for whom the impact is limited.

**CONCLUSION:**

Integrating clinical, demographic, genomic, and therapeutic data, to our knowledge, we have developed the first individualized risk-prediction model enabling personally tailored therapeutic decisions for patients with NDMM.

## INTRODUCTION

Clinical outcomes in newly diagnosed multiple myeloma (NDMM) have significantly improved during recent years because of the introduction of novel therapeutic agents.^1,2^ However, considerable heterogeneity remains, with a subset of patients only marginally benefitting from newer therapies, reflected in persisting short survival, while others experience decades of disease-free survival even with limited therapy.^3^ Proposed fluorescence in situ hybridization (FISH)–based updates to the International Staging System (ISS) have aimed for a better definition of risk in NDMM.^4-6^ Despite some improvements, the application of these models in clinical practice has been limited by several factors, such as (1) there remains considerable patient-to-patient variability within the defined risk groups; (2) they define the relative risk of either progression or death for a group of patients with similar features, but are not developed to predict individual patient outcomes; (3) treatment is not included, limiting their ability to inform therapeutic decisions; and (4) they largely ignore several prognostically relevant genomic and time-dependent features.

**CONTEXT**

**Key Objective**
Is it possible to use genomics to expand biological classification and develop individualized prognostication in multiple myeloma (MM)?
**Knowledge Generated**
Leveraging an extensive repository of genomic drivers in a series of 1,933 patients with newly diagnosed MM, we expanded previous fluorescence in situ hybridization and gene expression–based models identifying 12 distinct biological groups. By integrating clinical, genomic, and treatment data and validating with the GMMG-HD6 trial, we created a predictive model for individualized risk in newly diagnosed MM (IRMMa) that demonstrated superior accuracy compared with existing prognostic methods (eg, International Staging System [ISS], revised [R]-ISS, and R2-ISS).
**Relevance *(S. Lentzsch)***
By integrating 20 highly relevant genomic features, IRMMa allows better identification of primary refractory and early progressive myeloma patients compared to current staging systems such as R-ISS and R2-ISS. IRMMa subsequently boosts overall survival prediction accuracy and could guide clinicians in adjusting for treatment and consolidation strategies.*
  *Relevance section written by *JCO* Associate Editor Suzanne Lentzsch, MD, PhD.

Recent whole-genome, whole-exome, and targeted sequencing studies have identified a number of recurrent and prognostic genomic features.^7-11^ Despite these advances, NDMM is still clinically classified based on FISH and gene expression profiling (GEP) models,^11-13^ reflecting difficulties in developing robust clustering and classification approaches that correct for the co-occurrence of different genomic features.^8^ In a disease as heterogeneous as multiple myeloma (MM), similar large-scale data integration approaches have the potential to identify distinct groups of patients predicted to benefit from particular treatments (eg, high-dose melphalan followed by autologous stem-cell transplantation [HDM-ASCT]).

In this study, we assembled a large training set (N = 1,933) and a validation set (N = 256) of patients with NDMM with available clinical, demographic, genomics, and therapeutic data, to develop a comprehensive genomic classification of NDMM and to develop, to our knowledge, the first individualized prediction model able to incorporate heterogeneous clinical and genomic information to predict an individual MM patient's response to given treatment options.

## METHODS

Key clinical and genomic features of both the training and the validation set are summarized in the Data Supplement (Table S1 and Fig S1, online only).^11,14,15^ The full analytical workflow and codes for the genomic classification and the prediction model for individualized risk in NDMM (IRMMa) are available in the Data Supplement (Methods and Data S1 and S2), and GitHub.^16^

## RESULTS

### MM Genomic Driver Landscape

**FIG 1.**
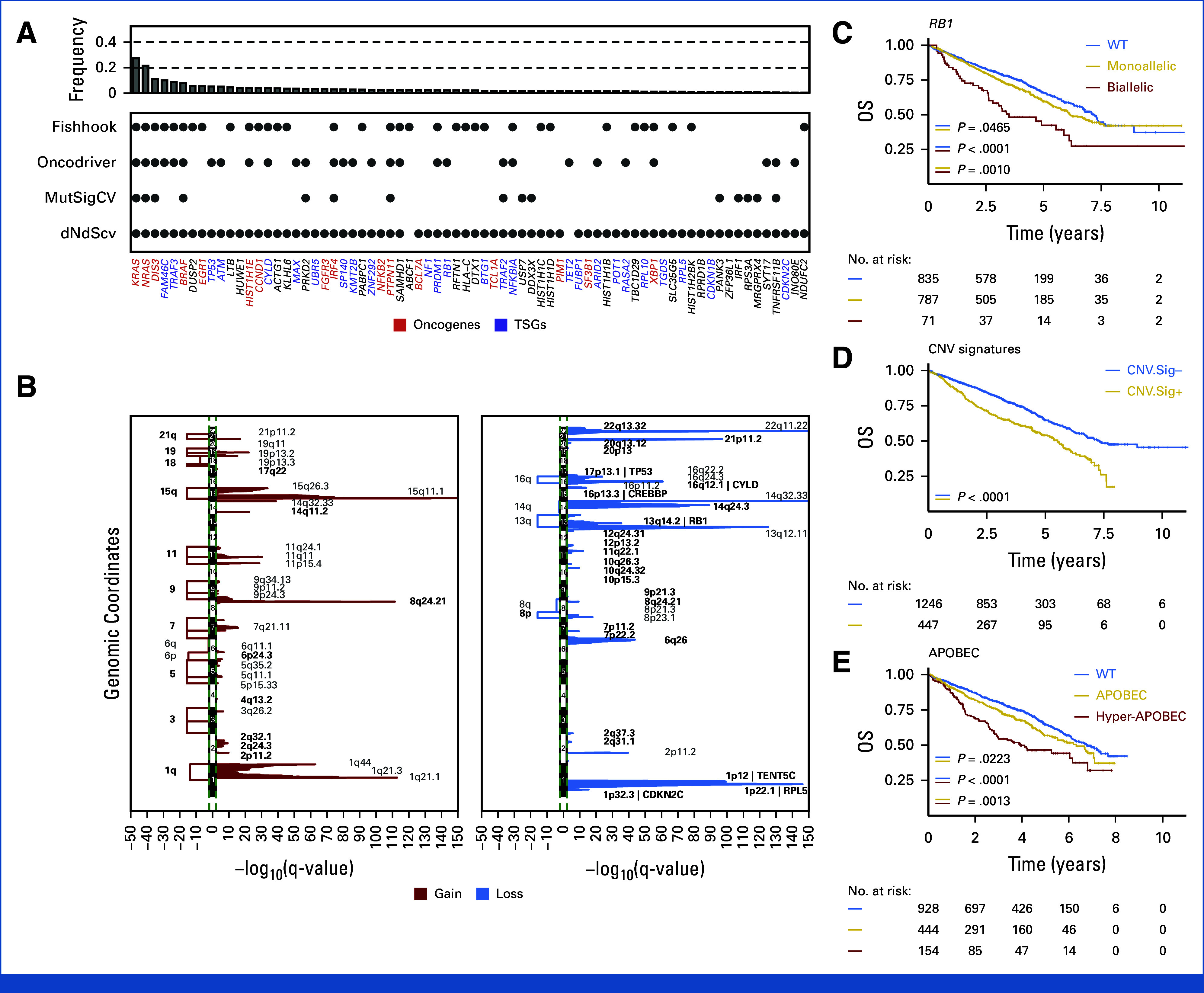
Genomic driver landscape in newly diagnosed multiple myeloma. (A) Driver genes significantly involved by single-nucleotide variants and indels using four different driver discovery tools (Fishhook, Oncodriver, MutSigCV, and dNdScv). X-axis label colors represent the COSMIC census annotation for each driver gene: red = oncogenes; blue = TSG; black = unknown. (B) Significant broad and focal copy-number changes detected by GISTIC: red = gain, blue = loss. (C-E) Kaplan-Meier curves for OS according to (C) *RB1* allelic status, (D) presence of chromothripsis-CNV.Sig, and (E) APOBEC activity. CNV.Sig, CNV signature; GISTIC, Genomic Identification of Significant Targets in Cancer; OS, overall survival; TSG, tumor-suppressor genes; WT, wild type.

Across 1,727 (89.3%) NDMM with available single nucleotide variants and indel calls, we identified 90 putative driver genes significantly enriched for nonsynonymous mutations, 10 of which have not been previously reported (Fig 1A; Data Supplement, Tables S2 and S3).^17-20^ In line with previous evidence,^8,11^ the most frequently mutated driver genes were *KRAS* (24.3%), *NRAS* (20.1%), *DIS3* (9.4%), *TENT5C* (8.6%), *BRAF* (7.8%), and *TRAF3* (6.6%). Seventy-nine percent of patients had at least one nonsynonymous mutation in at least one of the 90 driver genes.^8,11,21,22^ Interrogating the copy number variant (CNV) landscape, we found 88 loci recurrently involved by CNV: 34 focal deletions, five large deletions, 30 focal gains, and 19 large gains (Fig 1B; Data Supplement, Tables S2 and S4). Overall, at least one recurrent aneuploidy was observed in 77.8% of cases. Fifty-three percent of the patients had at least two large chromosomal gains on odd-numbered chromosomes and were defined as hyperdiploid (HRD).^23^

CNV analysis and clustering in MM has been historically difficult because large CNVs often affect multiple driver genes and Genomic Identification of Significant Targets in Cancer (GISTIC) peaks, making it harder to identify the relevant driver gene and the independence of minimally deleted/gained chromosomal regions.^3,8,11,24-26^ To define and correct for dependencies between different CNVs within the same chromosomes and avoid duplicates, we investigated for each GISTIC peak the impact of the CNV size and number of copies (ie, >three copies, here defined as amplification), the relationship with other GISTIC peaks, the impact on GEP, and clinical outcomes (Fig 1C; Data Supplement, Tables S4-S6, Figs S2 and S3, and Data S1). Large (>5 Mb) and focal (<5 Mb) CNV events involving multiple GISTIC peaks on 1q, HRD chromosomes, 8p, 16q, and 13q did not show any major differences compared with the focal, and therefore, different focal GISTIC peaks within each of those chromosomes were aggregated. Among the chromosomal amp, only 1q showed evidence of cumulative CNV effect on GEP.^11,22,27^ Overall, 32 tumor suppressor genes (TSG) had biallelic inactivation in 509 (32.8%) patients. Those most recurrently involved by biallelic events were *TRAF3* (7.9%), *CYLD* (4.7%), *TP53* (3.7%), *RB1* (4.1%), *MAX* (3.8%), *TENT5C* (3.3%), and *CDKN2C* (2.3%). Defining TSG involved by monoallelic and biallelic loss is relevant not only from a cell biology but also from the prognostic standpoint.^3,28,29^ In fact, among these events, biallelic loss of *RB1*, *TP53*, and *DNMT3A* were associated with a significantly shorter event-free survival (EFS) and overall survival (OS) when compared with monoallelic events (Data Supplement, Table S6 and Figs S2 and S3).

Chromothripsis is a complex structural variant strongly associated with poor outcome in NDMM.^10^ To capture this important feature, we used CNV signatures, described to accurately predict the presence of chromothripsis from both whole exome sequencing (WES) and targeted sequencing data.^7,30^ CNV signatures previously associated with chromothripsis were detected in 26.4% of cases and associated with both shorter EFS and OS (Fig 1D and Data Supplement Fig S2).

To complete our NDMM genomic profiling, we estimated APOBEC mutational signature contribution across patients with WES data (n = 1,526; 79%).^9,31,32^ Overall, 598 (39%) patients had clear evidence of APOBEC activity (SBS2 and SBS13), with the top 10th percentile (ie, >11%) defined here as hyper-APOBEC (n = 154; 10%). Patients with high APOBEC had a significantly worse outcome compared with those without (Fig 1E and Data Supplement Fig S2).^3,22^

### MM Genomic Classification

**FIG 2.**
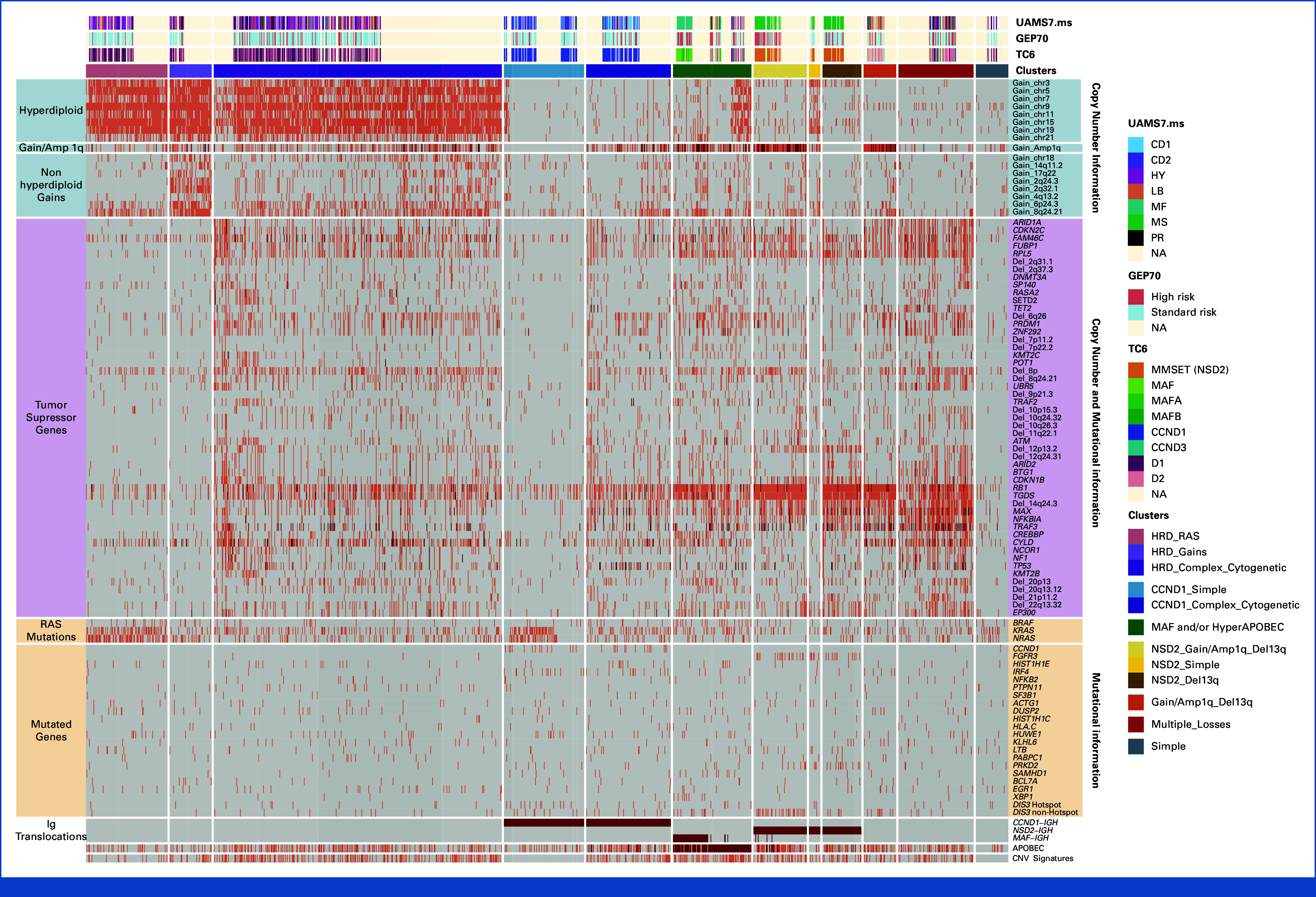
Newly diagnosed multiple myeloma genomic classification. The features defining 12 genomic clusters are defined, including GEP70 status, FISH-TC6, UAMS gene expression groups, IGH translocations, and all key mutational, copy number, and structural variant features reported in Figure 1. Gray: wild-type; red: single allele event or APOBEC; brown: biallelic event; hyper-APOBEC; 1q amplification, and IGH canonical translocations.

Although multiple genomic events and patterns of driver co-occurrence have been reported,^3,8,11,24-26^ MM molecular classification has not significantly changed over the past 15 years and still relies on FISH and GEP data (ie, the FISH-translocations and cyclin D [TC] and University of Arkansas for Medical Sciences [UAMS] classifications).^12,13,33-35^ This has been mostly driven by the difficulties in integrating different MM genomic drivers into clustering methods that correct for the multiple patterns of co-occurrences known to be common in MM. To address this historical issue, we interrogated 1,434 (74%) patients with available data on all the genomic drivers described and integrated above, implementing three different approaches: (1) pairwise analysis between each single genomic event; (2) higher-level interactions (ie, hierarchical Dirichlet process) combining genomic events with strong patterns of co-occurrence in the pairwise approach; and (3) reporting TSG as wild type, monoallelic, and biallelic loss. Overall, independently of established immunoglobulin translocations and HRD, two additional genomic patterns were observed (Fig 2; Data Supplement, Methods, Figs S4 and S5, and Tables S7 and S8). The first was characterized by the presence of RAS pathway mutations (*NRAS*, *KRAS*, and *BRAF*) and low prevalence/absence of recurrent aneuploidies and biallelic events; the second was mutually exclusive of the first and had a significantly higher prevalence of genomic complexity co-occurring with multiple large deletions, biallelic events, chromothripsis-CNV signatures (CNV.Sig), high APOBEC, and 1q gain/amp. In line with its complex genomic profile, the second group had a shortened OS compared with the first (*P* = .002; Data Supplement, Fig S6A). Integrating these findings into the FISH-TC classification, we were able to divide NDMM into 12 main MM genomic clusters (Fig 2; Data Supplement, Figs S6 and S7, Data S1, and Methods).^12,35^ HRD cases without Ig translocations (previously assigned to the D1 and D2 TC groups) were subdivided in three genomic groups. The first, named HRD_RAS (9%) was characterized by HRD, RAS mutations, and a simple genome lacking multiple aneuploidies. By contrast, the second cluster (HRD_Complex; 32%) was enriched for aneuploidy and chromothripsis-CNV.Sig. The third group (HRD_Gains; 4.6%) contained simple genomes, with an absence of RAS mutations and the presence of large gains on chromosomes 2, 4q, 6p, 8q, and 17q. The previous TC1 group, harboring t(11;14), was divided in two: CCND1_Complex (9.4%) and CCND1_Simple (8.9%). In the first, *CCND1* translocation co-occurred with several deletions, 1q gain/amp, and chromothripsis-CNV.Sig, reflecting a complex genomic profile. By contrast, CCND1_Simple had either mutation in the RAS pathway genes, *IRF4*, or a concurrent HRD profile, without features associated with genomic complexity. Interestingly, patients in CCND1_Simple had better survival compared with those in CCND1_complex (*P* = .028; Data Supplement, Fig S6B), providing increased resolution of t(11;14) biology, currently considered uniformly low- or intermediate-risk. The previous TC4 group harboring t(4;14) was divided into three: in NSD2_1q_13q (5.9%), t(4;14) co-occurring with del13q, 1q gain/amp, and nonhotspot mutations in *DIS3* (ie, D479, D488, and R780),^36^ NSD2_13q (4.3%) had t(4;14) with del13q, but not 1q gain/amp, while in NSD2_Simple (1.2%), t(4;14) was not associated with either 1q gain/amp or del13q, had large chromosomal gains, but a low-complexity genome. As the co-occurrence of del13q and 1q gain/amp was a high prevalence in the data set and was independent from the simple and complex genomic patterns, an additional genomic cluster was created including patients carrying these two genomic drivers without *NSD2* translocations (1q_13q; 3.6%). Interestingly, while genomically distinct, the outcomes of these four clusters did not differ (Data Supplement, Fig S6C). The previous TC5 group harboring *MAF*/*MAFB* translocations showed a complex genomic profile associated with high APOBEC. These patients were combined with high APOBEC patients without *MAF/MAFB* translocations, considering the similarities in their overall genomic profile and clinical outcomes (MAF_APOBEC; 8.7%; Data Supplement, Fig S6D). Finally, the remaining cases previously classified as either D1 and D2 without HRD and Ig translocations were divided into two further clusters: one with multiple aneuploidies and chromothripsis-CNV.Sig (Multiple_Losses; 8.3%) and one with a low complexity genome (Simple; 3.6%). Changes were also correlated with the UAMS GEP-based classification (Data Supplement, Data S1),^13^ with the high-risk proliferation (PR) group distributed across two complex groups: HRD_Complex (54%) and Multiple_Losses (14%; Data Supplement, Fig S7). Overall, this new genomic classification allowed us to better decipher the clinical and biological heterogeneity seen in comparison with both the TC and UAMS groups.

### IRMMa

**FIG 3.**
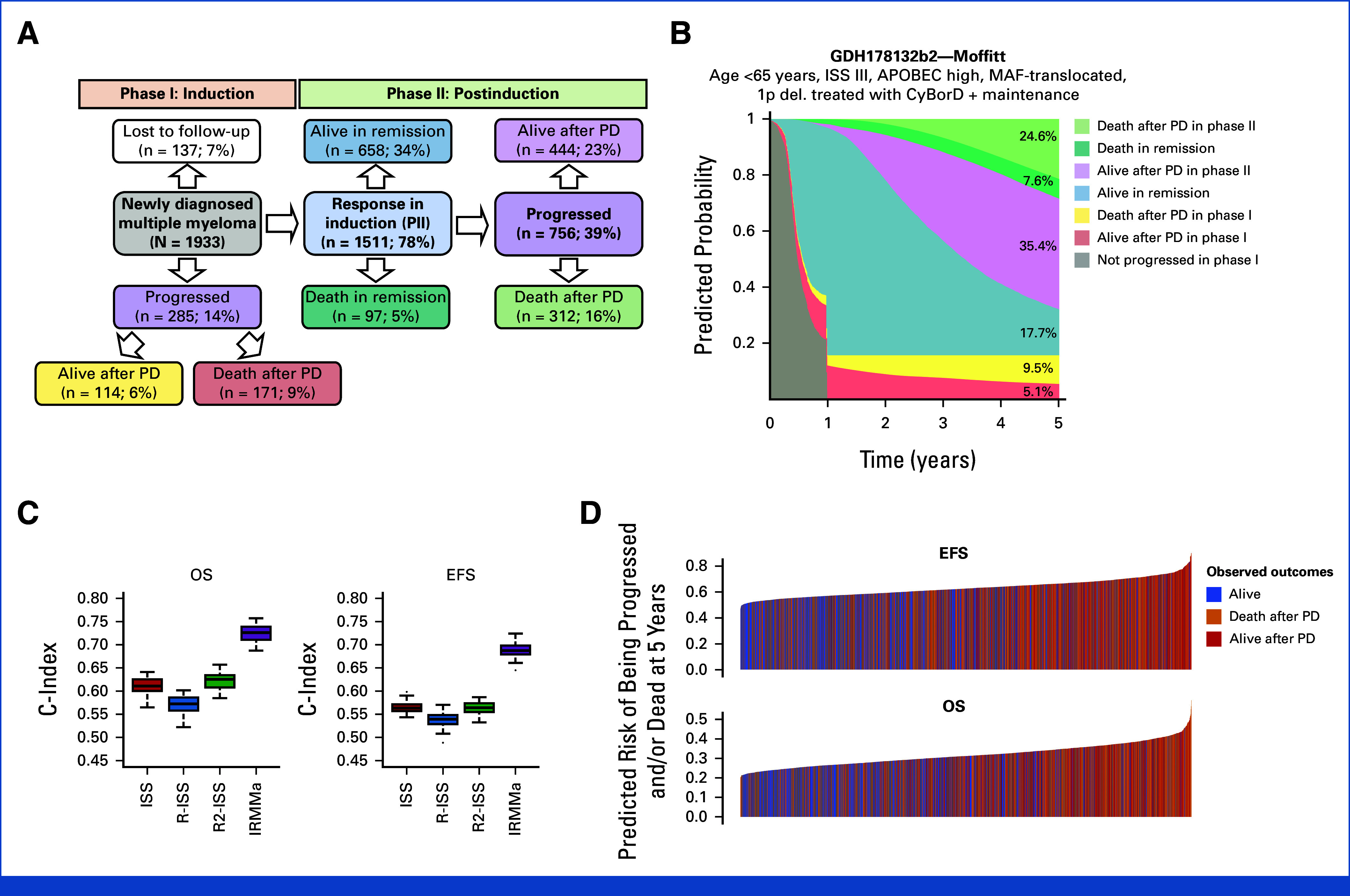
IRMMa. (A) Multistate model of a patient with NDMM. The six colored shapes correspond to different stages across the two phases (phase I: induction and phase II: postinduction), with different possible transitions (arrows). The number and percentage in each shape corresponds to the total number of patients who entered during the study follow-up. Among lost to follow-up in phase I group, 66/137 (48%) died in phase I for other causes. IRMMa was developed using NCNPH. (B) Sediment plot showing the risk over time for a single patient with NDMM from diagnosis to 5 years. The patient's clinical and genomic profile was predicted as high risk with a predicted probability be alive and in remission of 17.7% in line with the clinical and genomic high-risk presentation. (C) Boxplots comparing c-index for ISS, R-ISS, R2-ISS, and IRMMa for OS and EFS. Boxplots are generated using stratified cross-validation (5-fold × 10-random-repeats = 50 splits). As expected, R-ISS showed a lower accuracy than ISS and R2-ISS. This is because R-ISS was developed with a focus on high sensitivity, which means it is good at identifying patients with high-risk NDMM. However, this focus on sensitivity comes at the cost of specificity, which means that it is more likely to incorrectly classify high- and intermediate-risk patients as intermediate- and low-risk, respectively. (D) Model performance comparing predicted risk of progression (EFS) or death (OS) and observed (ie, bar colors). EFS, event-free survival; IRMMa, individualized prediction model for newly diagnosed multiple myeloma; ISS, International Staging System; NCNPH, neural Cox nonproportional hazards; NDMM, newly diagnosed multiple myeloma; OS, overall survival; PD, progression disease; R-ISS, revised international staging system.

**FIG 4.**
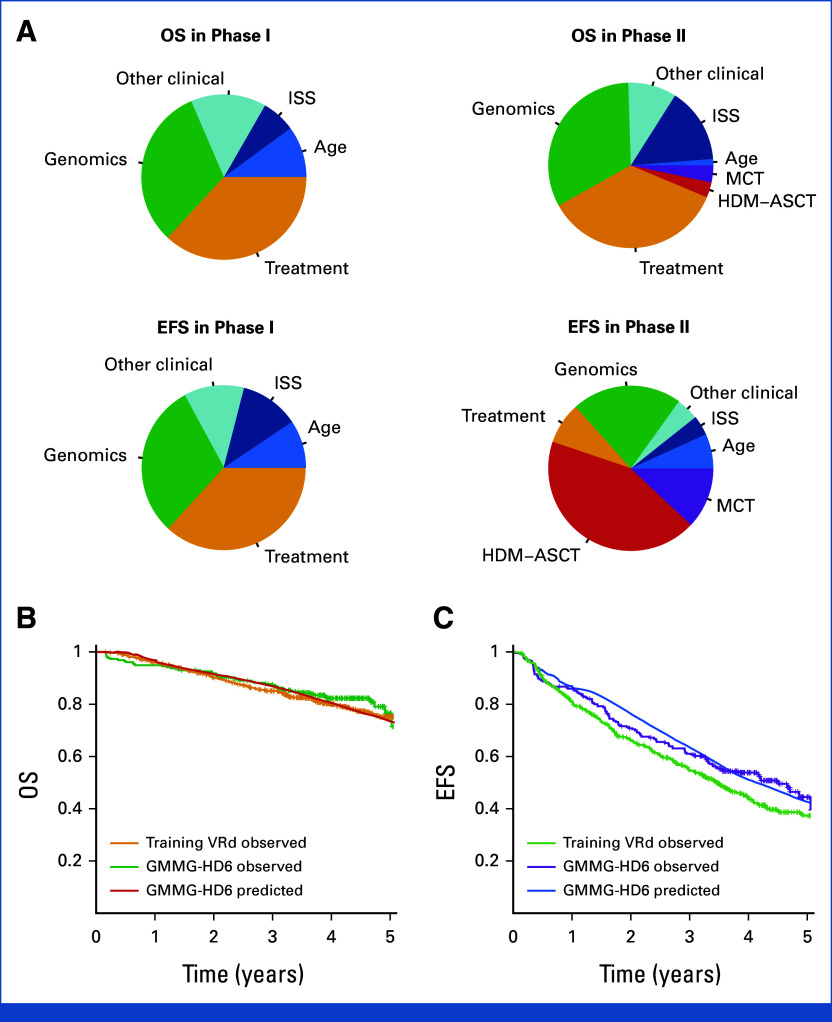
IRMMa anatomy. (A) The relative weight of age, ISS, genomics, treatment, other clinical (ie, ECOG, sex, race, and LDH level), HDM-ASCT, and maintenance/continuous treatment on our prediction model (IRMMa) for each of the multistate phase. (B and C) Predicted and observed outcome for the GMMG-HD6 cohort. The observed clinical outcome for patients included in the training treated with VRd + HDM-ASCT + MCT was also included. ECOG, Eastern Cooperative Oncology Group; HDM-ASCT, high-dose melphalan followed by autologous stem-cell transplantation; IRMMa, individualized prediction model for newly diagnosed multiple myeloma; ISS, International Staging System; LDH, lactate dehydrogenase; MCT, maintenance/continuous treatment; VRd, bortezomib, lenalidomide, and dexamethasone.

After a median follow-up of 43 months, 1,041 (53.8%) patients relapsed, 285 (14.7%) of which occurred during induction (phase I). Overall, 646 (33%) patients died, 483 (24%) of which due to MM. Integrating clinical, demographic, genomic, and treatment data, we developed IRMMa to predict individualized risk for OS and EFS (c-index 0.726 and 0.687, respectively; Figs 3A and 3B; Data Supplement, Methods and Data S2).^37^ IRMMa's accuracy was significantly higher than all existing prognostic models: ISS (EFS, 0.563; OS, 0.61), revised (R)-ISS (EFS, 0.539; OS, 0.572), and R2-ISS (EFS, 0.563; OS, 0.625; Figs 3C and 3D; Data Supplement, Fig S8). Among all 132 genomic features tested, we found 20 to improve model accuracy significantly, including 1q21 gain/amp, *TP53* loss, t(4;14; *NSD2*;*IGH*), chromothripsis-CNV.Sig, hyper-APOBEC, and deletions on 1p (Fig 4A; Data Supplement, Fig S9 and Table S9). Genomics emerged as important in predicting patients who progressed during the induction (ie, refractory NDMM) and significantly boosted accuracy for OS. Among the different clinical features tested, age and ISS were the most important for the model accuracy. By contrast, the impact of sex, Eastern Cooperative Oncology Group, race, and lactate dehydrogenase (LDH) was limited. The first-line treatment choice emerged as a key determinant of risk, suggesting that effective therapies may modify the risk associated with clinical and genomic variables and may thus have a different impact in the context of individual patients (Data Supplement, Fig S9). Importantly, because IRMMa was built as a multistate model, we could integrate and quantify the impact of time-dependent features such as HDM-ASCT and maintenance/continuous treatment. This is a key methodologic improvement compared with previous models,^4,5^ allowing to correct and quantify the clinical impact of these two postinduction treatments. In line with the most recent literature,^38-42^ HDM-ASCT and maintenance/continuous treatment had a major impact on EFS in phase II and a smaller one on OS in phase II (Fig 4A; Data Supplement, Figs S9, S10A and S10B). Overall, these data demonstrate the importance of including genomic and treatment features in predicting NDMM patients' OS and EFS, respectively. As a representative example, a patient with a low-risk genomic profile may experience short EFS because of a lack of exposure to effective therapy for their particular disease subset. OS for the same patient may, however, not be affected because of the impact of varying and potentially more effective subsequent therapies. By contrast, a patient with a high-risk genomic profile was generally resistant to most therapies, reflecting both short EFS and OS.

Although the inclusion of each feature improved the model, IRMMa has been developed as a flexible tool able to predict outcomes with incomplete data. Specifically, because genomic profiling is only rarely performed in the current clinical practice for NDMM, IRMMa performances were tested without genomic data. Despite this, IRMMa still outperformed ISS, R-ISS, and R2-ISS with OS and EFS (Fig 3C; Data Supplement, Figs S10C and S10D).

Finally, the IRMMa model performance was validated on the 256 patients enrolled in the GMMG-HD6 trial with available genomic data. Overall, IRMMa showed a higher accuracy for EFS and OS compared with ISS, R-ISS, and R2-ISS in predicting clinical outcomes (Data Supplement, Data S2). Furthermore, to validate the model accuracy, we leveraged IRMMa as a knowledge bank^43^ to predict outcomes in the GMMG-HD6 cohort, observing high concordance between predicted risks and observed outcomes (OS and EFS c-index 0.65 and 0.58, respectively; Figs 4B and 4C).

### Treatment Variance in NDMM

**FIG 5.**
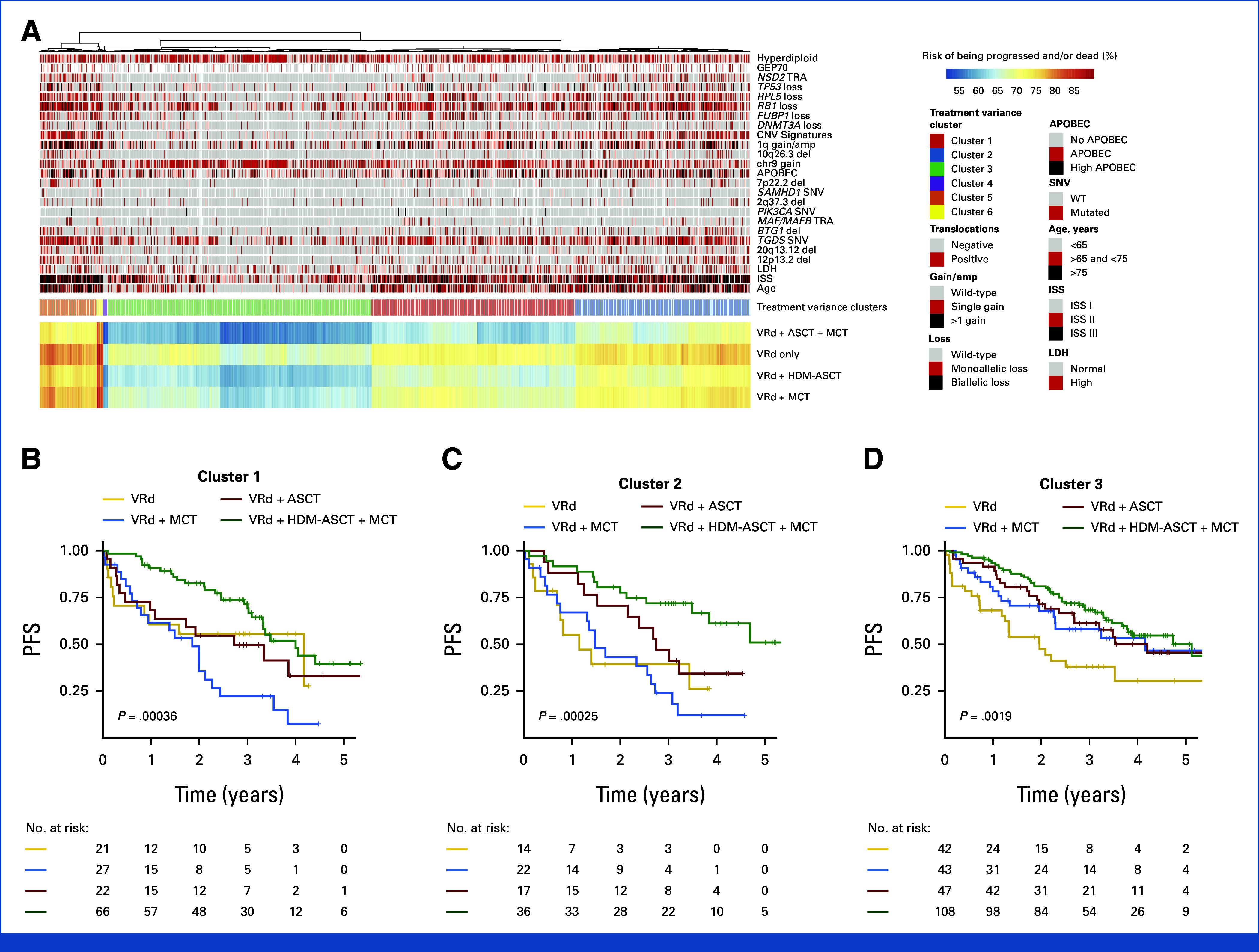
Predicted treatment variance in patients with newly diagnosed multiple myeloma treated with VRd. (A) Heatmap showing the predicted treatment variance across 1,933 patients in case of treatment with VRd ± HDM-ASCT ± MCT. (B-D) Observed probability to be alive and in remission (PFS) across the treatment variance groups defined in (A). To correct for HDM-ASCT and maintenance/continuous treatment, time was calculated from the end of induction to the last follow up (ie, phase II). Statistical differences between all different treatment groups within each cluster was estimated by log-rank test and reported in the Data Supplement (Table S10). HDM-ASCT, high-dose melphalan followed by autologous stem-cell transplantation; IRMMa, individualized prediction model for newly diagnosed multiple myeloma; ISS, International Staging System; LDH, lactate dehydrogenase; MCT, maintenance/continuous treatment; PFS, progression-free survival; SNV, single nucleotide variant; VRd, bortezomib, lenalidomide, and dexamethasone; WT, wild type.

As a key innovation compared with other prognostic models for NDMM,^4,6,41^ IRMMa also allows prediction of the risk for each state according to which therapy is administered, after correction for key genomic and clinical features. Specifically, we identified eight induction strategies in our series, on the basis of immunomodularity agents (IMIDs), proteosome inhibitors (PIs), and chemotherapy (eg, cyclophosphamide, low-dose melphalan, platinum-based regimens), and four possible postinduction strategies: observation, HDM-ASCT, HDM-ASCT + maintenance/continuous treatment, and maintenance/continuous treatment without HDM-ASCT, for a total of 32 possible treatment courses. The risk of not having progressed and/or being dead at 5 years (progression-free survival [PFS]) was predicted for each patient in each possible treatment course. The PFS difference between courses within the same patient was defined as treatment variance. To evaluate meaningful patterns in the context of the current therapeutic landscape, we explored the impact of HDM-ASCT and maintenance/continuous treatment after induction with bortezomib, lenalidomide, and dexamethasone (VRd; Fig 5A; Data Supplement, Table S10).^42^ Integrating predicted outcomes and treatment variance for all four possible treatment combinations (ie, VRd ± HDM-ASCT ± maintenance/continuous treatment), we identified six main clusters. In cluster 1 (n = 554), the intensive combination of HDM-ASCT plus maintenance/continuous treatment was effective in converting unfavorable outcomes into favorable ones (Fig 5B). In cluster 2 (n = 476), patients had a high treatment variance with significant benefit from receiving HDM-ASCT and a relatively small advantage in receiving maintenance/continuous treatment (Fig 5C). In cluster 3 (n = 717), patients were usually age younger than 65 years with low ISS and low genomic complexity. In line with this presentation, any consolidation strategy provided an advantage, with no significant difference between HDM-ASCT and other maintenance/continuous treatments (Fig 5D). The other three groups included a smaller number of patients, with cluster 4 (n = 13) associated with favorable outcomes independent of the postinduction strategy. Cluster 5 (n = 155) was enriched for patients with high-risk genomic and clinical features, and poor outcomes, partially improved by HDM-ASCT. Clusters 6 (n = 18) included a small number of patients with aggressive clinical and genomic features and limited treatment variance. Patients enrolled in the GMMG-HD6 were mostly assigned to clusters 1 and 3, suggesting that a fraction of patients might have had favorable outcome even without HDM-ASCT (Data Supplement, Fig S11).

There were significant differences in treatment variance among the 12 genomic groups, with each having predictable sensitivity to different therapies (Fig 2; Data Supplement, Figs S12 and S13 and Table S11). Specifically, groups with less complex genomes (HRD_RAS, HRD_Gains, CCND1_Simple, and Simple) tended to be grouped in cluster 3, suggesting high sensitivity to VRd with and without HDM-ASCT. CCND1_Complex, HRD_Complex, and Complex were mostly divided between cluster 1 and cluster 3. NSD2_HRD, MAF_APOBEC, and 1q_13q were divided across cluster 1 and cluster 2. Finally, NSD2_1q_del13q, and NSD2_13q were mostly in cluster 2 and cluster 5, suggesting potential sensitivity to intensification with HDM-ASCT.

IRMMa is available for estimating individualized risk and treatment variance of NDMM as an online tool for the research community (IRMMa Risk Calculator^44^).

## DISCUSSION

In this study, we leveraged a large and diverse data set of patients with NDMM to identify key genomic drivers and propose a more comprehensive genomic classification able to better capture heterogeneity among defined molecular subgroups creating opportunities to better decipher clinical heterogeneity and treatment sensitivity.^12,13,34,35^ Compared with recent efforts,^3,8,11,24-26^ our study has three key advantages: (1) the larger sample size; (2) the analytical workflow that takes into account more genomic drivers, multiple confounders, and patterns of co-occurrence, which were partially overlooked in previous works; and (3) unlike previous efforts that primarily focus on individual driver events, our new classification system emphasizes the examination of genomic patterns of co-occurrence (eg, complex *v* simple).

Although recently proposed prognostic models, such as R-ISS and R2-ISS, can identify a subgroup of high-risk patients,^4,41^ they are not corrected for different treatment approaches and are not designed to predict patient-level individual risk. Integrating key features defined in our genomic classification together with clinical, demographic, and treatment data, we leveraged deep neural networks to develop, to our knowledge, the first prediction model for individualized risk in NDMM patient outcomes (ie, IRMMa). Compared with previous prognostic models, IRMMa has several key advantages. First, IRMMa integrates genomic features selected according to their prognostic relevance when corrected for clinical, demographic, and treatment features. The inclusion of 20 highly relevant genomic features significantly improves the IRMMa ability to identify primary refractory and early progressive patients and boosts accuracy for OS, confirming the need for expanded genomic characterization in NDMM prognostication. Second, IRMMa allows the estimation of the risk of progression or death for an individual patient with NDMM, adjusting for treatment and consolidation strategies. HDM-ASCT and maintenance/continuous treatment have been shown to significantly improve EFS,^38,40-42^ but because of their time-dependent nature, they have never been considered in the development of previous prognostic models (R-ISS and R2-ISS). The IRMMa multistate design allowed the inclusion of these features, improving the overall accuracy for EFS. Furthermore, the ability to capture each patient's specific treatment variance represents a critical tool that can help to select the most effective therapy and to avoid overtreatment where it adds little to no benefit. Of relevance, IRMMa can be relevant for identifying patients who do, or do not, benefit from HDM-ASCT. Several randomized phase III trials have explored the advantage of HDM-ASCT as a consolidation strategy after IMIDs and/or PIs.^38,40,42,45,46^ In most studies, HDM-ASCT has been associated with an advantage in PFS overall, but not in OS. These observations raise clinically important questions on how to counsel patients with NDMM, particularly in the future era of novel effective immunotherapies. Finally, the implementation of IRMMa necessitates the inclusion of ISS, age, and treatment as mandatory features. Thus, even with a reduced concordance rate, IRMMa has the capability to generate estimates even in the absence of genomic data, surpassing the predictive accuracy of R2-ISS, R-ISS, and ISS, presenting an opportunity to improve predictions without the availability of comprehensive genomics.

**FIG 6.**
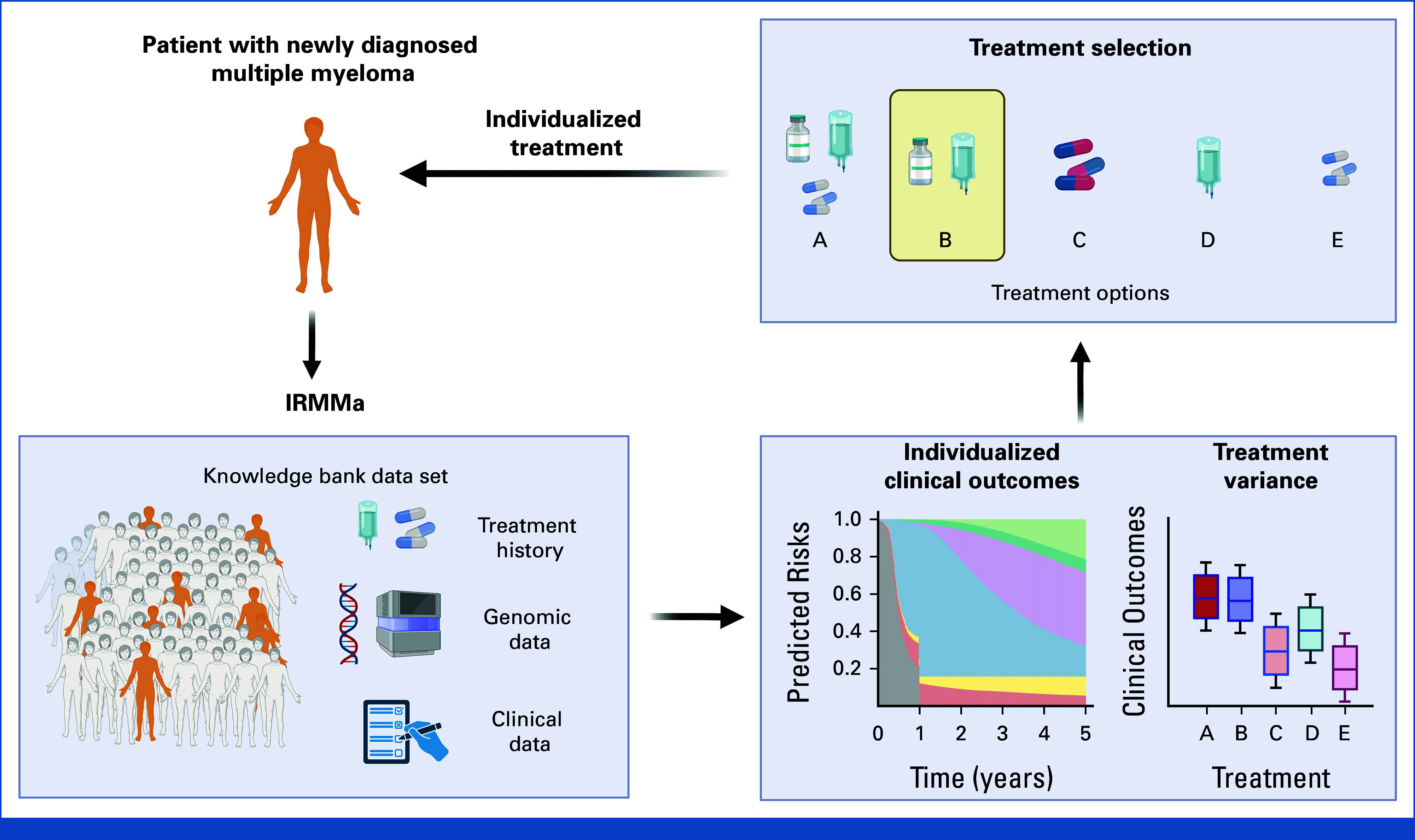
Figure summarizing IRMMa potential integration into clinical applications. The figure was generated using BioRender. IRMMa, individualized prediction model for newly diagnosed multiple myeloma.

Overall, IRMMa has some limitations: (1) the sample size used in the training set was smaller than the one used to develop R-ISS and R2-ISS; (2) IRMMa was built using genomic data from a single bone marrow site and does not consider the potential impact of genomic drivers at different anatomic sites (ie, spatial heterogeneity).^47,48^ Future integration of bone marrow and liquid biopsy approaches might further improve IRMMa's performances and resolution. (3) Finally, the current IRMMa model cannot provide estimates for new agents (eg, anti-CD38 antibodies) and distinct time-dependent features (eg, minimal residual disease), as these data are not available for sufficiently large cohorts yet. However, in contrast to other models, such as the R-ISS, IRMMA has been built as a flexible and knowledge-driven model that can be grown over time by integrating additional genomic drivers, novel treatments, and their effect on treatment variance (Fig 6).

In conclusion, IRMMa represents an innovative opportunity to better investigate the heterogeneity of patients with NDMM, which is currently oversimplified, to improve our understanding of outcomes in both previous and future clinical trials.

## Data Availability

CoMMpass genomic data are available on dbGap: phs000748.v1.p1. UAMS genomic data are available on EGA: EGAS00001003223. The MGP data set is available in the European Genome-Phenome Archive under accession numbers EGAS00001001147, EGAS00001000036, and EGAS00001002859. Memorial Sloan Kettering Cancer Center myType: European Variation Archive with accession numbers PRJEB31370 (project) and ERZ807140 (analyses). AVATAR—Moffitt: Requests for access to the data used in this study can be submitted here at https://researchdatarequest.orienavatar.com/. GMMG-HD6: genomic data will be available on EGA EGAS00001007469. All R and Python codes used for this study can be found in the Data Supplement (Data S1 and S2) and on GitHub: https://github.com/UM-Myeloma-Genomics/GCP_MM.
